# The Influence of UV Light on Rheological Properties of Collagen Extracted from Silver Carp Skin

**DOI:** 10.3390/ma13194453

**Published:** 2020-10-08

**Authors:** Alina Sionkowska, Katarzyna Lewandowska, Katarzyna Adamiak

**Affiliations:** 1 Department of Biomaterials and Cosmetics Chemistry, Faculty of Chemistry, Nicolaus Copernicus University in Torun, Gagarin 7 street, 87-100 Torun, Poland; reol@umk.pl (K.L.); kadamiak@wellu.eu (K.A.); 2 WellU sp.z.o.o, Wielkopolska 280, 81-531 Gdynia, Poland

**Keywords:** collagen, fish skin, *Silver Carp*, rheology, UV irradiation

## Abstract

Acid soluble collagen (ASC) was extracted from *Silver Carp* fish skin. Collagen was dissolved in acetic acid at varying concentrations and its rheological properties were studied. Steady shear flow properties of collagen solutions at concentrations of 5 and 10 mg/mL were characterized using rheometry at 20 °C. Collagen solutions were irradiated with UV light (wavelength 254 nm) for up to 2 h and rheological properties were measured. All the collagen solutions showed a shear-thinning flow behavior. A constant viscosity region was observed after 1 h of UV irradiation, which showed that collagen molecules were fully denatured. A short treatment with collagen solution by UV (ultraviolet) light led to an increase in viscosity; however, the denaturation temperature of UV-irradiated collagen decreased. Depending on the time of UV treatment, collagen extracted from *Silver Carp* fish skin may undergo physical crosslinking or photodegradation. Physically crosslinked collagen may find applications in functional food, cosmetic, biomedical, and pharmaceutical industries.

## 1. Introduction

Collagen is a widely used material in the cosmetics, biomedicine, and food industries. However, native collagen is sensitive to high temperatures and UV treatment. UV irradiation of collagen molecules leads to several photochemical reactions [1,2,3,4,5,6,7,8,9,10,11]. Under UV irradiation of collagen, conformational changes were observed in collagen extracted from rat tail tendon [3,4,5,6,7,8]. The UV treatment of the collagen materials may influence the mechanical properties of the final collagen products [8,9,10]. Although there are some interesting studies regarding collagen alterations by UV irradiation, the molecular mechanisms behind the influence of UV light on collagen are still unknown. It has been shown that aromatic amino acids play the main role in the absorption of UV light by collagen molecules [11]. After absorption of UV light, a photochemical process in collagen may occur. It should be emphasized that the photochemical behavior of collagen may differ depending on the collagen origin and type, because the content of aromatic amino acids (phenylalanine and tyrosine) in the biopolymer chain may be different [12]. The whole collagen family encompasses 29 genetically distinct collagen types. The major ones involve type I (found in skin, tendon, and bone tissues), type II (cartilage), and type III (skin and vasculature) collagen [13,14,15]. In food and cosmetic applications, type I collagen is prevalently used. In food, mainly denatured collagen known as gelatin finds its use because it is much cheaper than native collagen [16].

Collagen has been traditionally isolated from land-based animal skins, e.g., cow and pig skin. For scientific research, mainly rat tail tendon collagen is employed because its denaturation temperature is around 40 °C [6]. Non-denatured collagen finds applications in cosmetics, biomedical, and pharmaceutical industries [12,16]. Denatured collagen, however, known as gelatin, is used not only in food but also in pharmaceutical and biomedical products [17].

As it was said before, collagen can be obtained from several animal tissues. Bovine collagen has been widely applied; however, its employment is burdened with the risk of bovine spongiform encephalopathy (BSE) and transmissible spongiform encephalopathy (TSE) development. Scientists are looking for safer alternatives, resources different from mammalian tissues—for example, collagen extracted from marine sources (fish skin and scales, marine sponges, or jellyfish umbrella). In the case of cosmetic creams containing collagen obtained from the marine sponge and those containing mammalian collagen, when comparing their impact on skin, it can be noticed that their effects on the skin pH, moisture, and sebum are comparable. This means that in cosmetic preparations, mammalian collagen can be successfully replaced with marine sponge collagen. The potential of marine collagen was discovered approximately 70 years ago and, from this time, research has contributed to learning about the structural and physicochemical features of many marine origin collagens. However, it is worth mentioning that collagen properties may vary depending on the fish species. Fish collagen is commonly applied in food production and can replace mammalian collagen; however, its low denaturation temperature is usually its main disadvantage. The low denaturation temperature of fish collagen is a result of the significantly lower content of hydroxyproline in the polypeptide chain of fish collagen than in the chain of mammalian collagen. Collagen extracted from *Silver Carp* skin has attracted much attention in recent years due to its relatively high denaturation temperature in comparison to collagen from other fish species [18]. Although there are some papers regarding the properties of collagen from *Silver Carp,* to our best knowledge, the influence of UV irradiation on the rheological properties of such kinds of collagen has not been studied yet. Rheological properties of collagen are essential in several applications. In the case of cosmetic and food applications, its rheological behavior at different temperature values is essential to design a proper formulation and check its applicability under several conditions. Based on rheological study, one can design a product with properties which can be tailored to the desired use—for example, for tissue engineering purposes. Collagen gels can be used for studying cell–matrix mechanical interactions as well as developing tissue equivalents, the rheological properties of which are very important [19]. UV irradiation can be used as a sterilizing agent for several biomaterials, so it is very important to study the influence of UV light on collagen gels. The rheological behavior of several collagen solutions depends on the collagen concentration and temperature [20,21,22].

The concentrated solutions of synthetic and natural polymers are characterized by complex viscoelastic properties. Almost all these fluids show non-Newtonian behavior. For them, apparent shear viscosity (*η_a_*) changes with shear rate ($\dot{\gamma }$). If the apparent shear viscosity decreases with an increase in the shear rate, the fluid is shear-thinning, i.e., exhibits pseudo-plastic behavior—typical of many polymers [23,24,25]. The opposite trend, which occurs rarely, is named shear thickening (dilatant fluid). The shear stress curve obtained as a function of shear rate (so-called flow curve) at various temperature values allows calculation of the rheological parameters from different equations (e.g., Ostwald de Waele model, Cross model, Carreau model, and others) and recognition of the type of polymer flow behavior [24,25,26]. The knowledge of rheological properties of polymer solutions or fluids allows us to evaluate their final properties and enables the arrangement and optimization of the processing conditions.

The aim of this work was to study the rheological behavior of collagen obtained from *Silver Carp* skin before and after different times of UV irradiation. Knowledge about the rheological behavior of UV-treated collagen can be essential in the preparation of cosmetics and food products based on collagen from *Silver Carp* skin. Moreover, the possibility of sterilization of collagen gels for biomedical applications using UV treatment can be predicted. The UV-irradiated collagen gels can be further used for the preparation of wound healing materials.

## 2. Materials and Methods

### 2.1. Raw Materials and Collagen Preparation

Collagen was purchased from WellU sp. z.o.o, Gdynia, Poland. It was obtained by collagen isolation from *Silver Carp* skin. The skin fragments were removed manually and washed with chilled tap water to get rid of the adhering tissues. In the next stage, the material was disinfected with 3% hydrogen peroxide water solution, residues of which were further rinsed off. The purified skin was placed in a lactic acid solution and left for 3 days to extract the collagenous proteins. The thus obtained solution was pressed through the material, which allowed for collagen separation. The samples were then placed in polyethylene bags and stored at −25 °C until use.

The collagen solution was dialyzed against distilled water for 2 days and then lyophilized. After lyophilization, collagen gels were prepared in diluted 0.1 M acetic acid at the concentrations of 5 and 10 mg/mL. For prepared collagen gels, the rheological properties were measured. In the next step, collagen solutions were irradiated with UV light for different time intervals and again the rheological properties were measured.

### 2.2. Measurement of Rheological Properties

For the study of rheological properties, the solutions of collagen at the concentrations of 5 and 10 mg/mL were prepared. A rheological investigation was carried out on the prepared samples by means of a rotational viscometer, Bohlin Visco 88 (Malvern Panalytical, Malvern, UK), equipped with a heating system and a solvent trap kit. Steady-state viscosity curves were evaluated at different temperature values, i.e., 15–33 °C (samples were thermally equilibrated for 300 s), at a shear rate 19–250 s^−1^ range using a concentric cylinder. In order to evaluate the time dependence of the apparent shear viscosity, i.e., to observe whether thixotropy takes place, the flow measurements were performed in two steps: at increasing shear rates (from the minimum to the maximum shear rate value—upward curve) and their return to the maximum value at decreasing shear rates (downward curve). Additionally, the time-dependent viscosity for the collagen solution at two different, constant, and imposed shear rates, i.e., 90 and 250 s^−1^ at 16.5 and 25 °C, were determined.

Rheological behavior of the collagen solutions was analyzed with the well-known Ostwald de Waele model (Equation (1)) and Cross equation (Equation (2)) to determine the relationship between the apparent shear viscosity and the shear rate [24,25,26].

Ostwald de Waele model:

(1) $$\tau =k{\dot{\gamma }}^{n}\;\mathrm{and}\;\eta =\frac{\tau }{\dot{\gamma }}=k{\dot{\gamma }}^{n-1}$$

where *τ* is shear stress (Pa), $\dot{\gamma }$ is shear rate (1/s), *η* is shear viscosity (Pa·s), *n* and *k* are rheological parameters known as non-Newtonian index (dimensionless) and consistency index (Pa·s^n^), respectively. The value of *n* < 1 indicates the shear-thinning effect, and the value of *n* > 1 implies the shear-thickening behavior. If *n* is unity (*n* = 1), then *k* is identical to η and Equation (1) takes the form of Newton’s law.

Cross model:

(2) $$\eta =\eta_{\infty }\;\frac{\eta_{0}-\eta_{\infty }}{1+\lambda {\dot{\gamma }}^{m}}$$

where $\eta_{0}$ and $\eta_{\infty }$ are zero-shear viscosity and infinite viscosity (Pa·s), respectively, *λ* is the characteristic relaxation time (s), and *m* is a dimensionless constant corresponding to the fluid.

The temperature dependence of the apparent shear viscosity (*η*_a_) was determined with the Arrhenius equation [25,27]:

(3) $$\eta_{a}=A_{0}\exp(E_{a}/\mathrm{RT})$$

where *A*_0_ is a pre-exponential parameter and *E_a_* is the viscous flow activation energy.

### 2.3. UV Irradiation of Collagen Solution

Collagen solutions (the concentrations of 5 and 10 mg/mL) were irradiated using a UV lamp, ULTRAVIOL NBV 15, which emitted mainly UVC with 254 nm wavelength. Collagen solutions were irradiated at a distance of 5 cm from the UV lamp.

## 3. Results

### 3.1. Rheological Behaviour of Collagen Solutions and Gels

**Figure 1:**
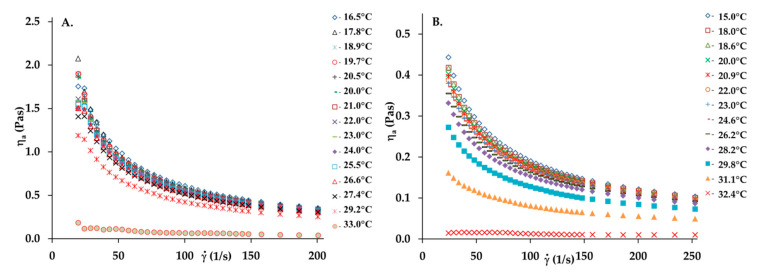
Viscosity curves of the collagen solutions: (**A**) 10 mg/mL, (**B**) 5 mg/mL in temperature range 15 to 33 °C.

Figure 1 reports the viscosity curves of collagen solutions with concentrations of 5 and 10 mg/mL (collagen gel).

**Figure 2:**
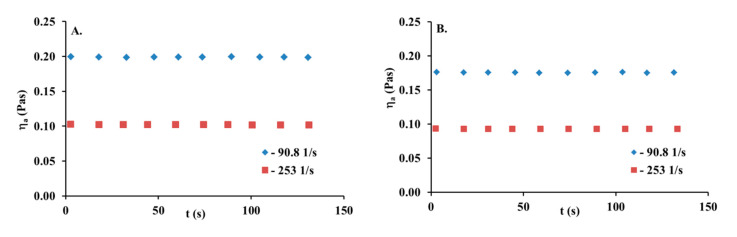
Time-dependent viscosity for 5 mg/mL collagen solution at two different and constant shear rates, (**A**) 16.5 °C, (**B**) 25.2 °C.

A collagen solution is characterized by the typical shear-thinning behavior of polymer solutions observed in the decrease in viscosity as the shear rate increases due to the progressive orientation and disentanglement of the chains. The apparent viscosity of the collagen solution is heightened with increasing concentrations. While the concentration of the collagen solution increases, the viscosity curves present a more pronounced shear-thinning behavior. This is caused by an increase in collagen macromolecule interactions, leading to the increase in the entanglement of the chains and more pronounced non-Newtonian behavior. The time-dependent apparent viscosity at the constant shear rate value is another important characteristic of the non-Newtonian behavior of polymer solutions [24]. Some of them are rheologically unstable fluids. Such solutions are classified as thixotropic and rheopectic fluids. For thixotropic ones, viscosity decreases in time, while the rheopectic fluid viscosity increases. Figure 2 shows the apparent viscosity and lapsed time relationship for a 5 mg/mL collagen solution at different, constant, and imposed shear rates. As can be seen, the constant and uniform viscosity value is observed in the time of the experiment (131 s). Thus, this collagen can be considered as rheologically stable.

### 3.2. The Ostwald de Waele and Cross Models

The experimental data of the collagen solutions used in this study were fitted with the Ostwald de Waele model (Equation (1)) and Cross model (Equation (2)), with R^2^ values ranging from 0.963 to 0.999 and from 0.994 to 1.00, respectively. The corresponding rheological parameters and regression coefficients (R^2^) are presented in Table 1. Noteworthy is the fact that all the *n* values from the Ostwald de Waele model were less than 1, indicating deviation from Newtonian behavior (shear-thinning behavior). An increase in the collagen solution concentration to 10 mg/mL causes a pronounced decrease in the *n* value from 0.39 to 0.26 at 18 °C. Such low *n* values may suggest the high association degree and collagen molecule entanglement in the solution. For all the collagen solutions, the temperature rise by 15 °C only slightly influences the *n* parameter (e.g., *n* increases from 0.39 to 0.43 in the collagen solution at the concentration of 5 mg/mL). The consistency index (*k*) values increase with an increase in the collagen solution concentration but insignificantly decrease as the temperature increases from 15 to 25 °C. As shown in Equation (1), the *k* parameter value presents a direct relationship with viscosity; it can be used to represent the viscosity characteristics of fluids under certain conditions. Therefore, the *k* results are as expected because the temperature will normally lower the viscosity of fluids. However, viscosity will increase together with a rise in concentration.

Moreover, both *η_0_* and *η_∞_* values increase simultaneously with the collagen concentration. The *η_0_* value is directly related to the number of interactions between the polymer macromolecules and the solvent [28,29]. Thus, the increase in the *η_0_* values together with an increase in the collagen solution concentration confirm that the development of a temporary network or the number of entanglements of collagen molecules in the solution increased. With the increased temperature, the *η_0_* values decreased, especially at temperatures ranging from 25 to 31 °C. This can be explained by the increase in the molecules’ mobility and the interactions between those which were partly destroyed.

### 3.3. The Effect of Temperature and the Viscous Flow Activation Energy

**Figure 3:**
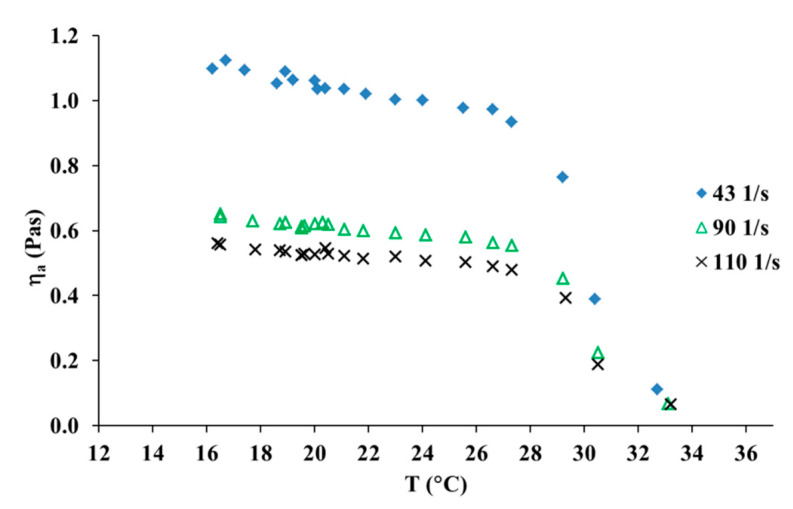
Apparent shear viscosity versus temperature for 10 mg/mL collagen solution at three different shear rates.

The effect of temperature on the apparent viscosity of collagen solutions was investigated. It can be observed that temperature showed a weak effect on the apparent viscosity, which decreased slightly with an increasing temperature range from 15 to 28 °C, as it has been shown in Figure 3. The increase in temperature from 30 to 32 °C caused a large reduction of the apparent viscosity value. Thus, the results show that collagen molecules are fully denatured at 32 °C.

The value of the viscous flow activation energy reflects the sensitivity of polymer solutions towards temperature. For the collagen solution, *E_a_* values were determined using Equation (3) at different shear rates (in the temperature range between 15 and 28 °C) and are tabulated in Table 2. As can be observed, the values of viscous flow activation energy decrease with an increasing shear rate and collagen concentration. This behavior can be explained by the entanglement formation in the polymer solution. At a higher shear rate ($\dot{\gamma }\;$≥ 90.9 s^−1^), the entanglement density between collagen molecules was independent of shear rate. The *E_a_* value is practically constant in this range. Moreover, the calculated *E_a_* values indicated that the collagen solution exhibited the low sensitivity of viscosity to temperature changes in the range of 15–28 °C.

### 3.4. The Effect of UV Irradiation on the Collagen Solution

**Figure 4:**
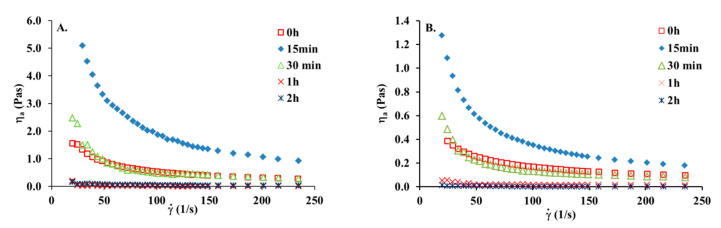
Viscosity curves of the collagen solutions: (**A**) 10 mg/mL, (**B**) 5 mg/mL at 20 °C before and after various times of UV irradiation.

Figure 4 shows viscosity curves for the collagen solution before and after various times of UV irradiation. It can be seen that after a short time of U irradiation, an increase in viscosity is observed. After 15 min of UV irradiation viscosity increased almost three times. This may suggest that UV irradiation causes the crosslinking of collagen molecules. In general, after absorbing a photon, the electronically excited molecule either undergoes a photochemical process, such as fragmentation, rearrangement, or photoaddition, or it will lose its energy via one of several photophysical ways (fluorescence, phosphorescence, internal conversion). It seems that short action of UV leads to photoaddition which is manifested by an increase in viscosity. After 30 min of UV treatment, the viscosity of the irradiated collagen solution is still higher than that for collagen without irradiation. After one hour of irradiation, the collagen solution lost its viscous properties. This suggests that after one hour of UV irradiation, collagen molecules are fully denatured. Prolonged UV treatment of collagen solution leads to the breaking of hydrogen bonds and fragmentation of macromolecules. When collagen solution is irradiated for 2 h and longer than 2 h, collagen is denatured and no crosslinking reaction appears. This clearly suggests that irradiation for more than two hours may lead only to the degradation of collagen chains, without any influence on rheological properties. The viscosity of the collagen solution with a concentration of 10 mg/mL is higher than that for the collagen solution with a concentration of 5 mg/mL. However, the rheological behavior after UV irradiation is similar to irradiated collagen solution with a concentration of 5 mg/mL After 15 min of UV irradiation, viscosity increased almost three times, from around 1.7 to 5.3 Pas.

Table 3 presents the alteration of a rheological parameter after UV irradiation of the collagen solution. The values of *n* remained at a similarly low level after UV irradiation to those before UV treatment and exhibited an obvious decrease from 0.27 to 0.13 with increasing temperature. Moreover, the values of *η*_0_ for the collagen solution increased from 2.742 Pas before irradiation to 20.04 Pas, implying the formation of a polymer network under the influence of a short time of exposure to UV treatment.

**Figure 5:**
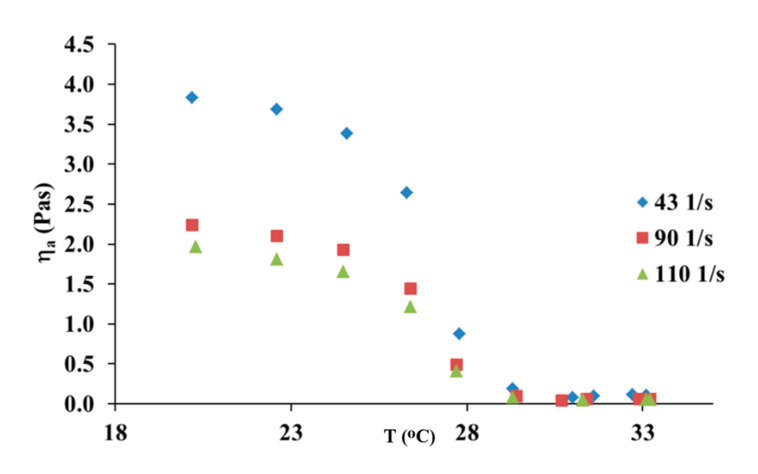
Apparent shear viscosity versus temperature for 10 mg/mL collagen solution at three different shear rates after 15 min of UV irradiation.

In Figure 5, one can see the influence of UV irradiation on the apparent shear viscosity of the collagen solution. As one can see, the rise in temperature from 25 to 29 °C caused a large decrease in the apparent viscosity. Therefore, the denaturation temperature decreased after 15 min of UV irradiation of the collagen solution. The UV-irradiated collagen molecules are fully denatured at 30 °C, whereas for collagen molecules which had not been treated with UV light, the molecules are fully denatured at 32 °C. This suggests that UV irradiation leads to partial cleavage of hydrogen bonds responsible for the ternary structure of collagen.

Table 4 shows that the activation energy was changed in the viscous flow of the collagen solution after 15 min of UV irradiation. For the collagen solution before exposure to UV irradiation, *E*_a_ values are significantly lower (Table 2) than after UV irradiation (Table 4). Moreover, the values of viscous flow activation energy increase with an increasing shear rate in the range between 43.3 and 110 s^−1^. This fact may suggest that photocrosslinking reactions occur in the collagen solution and new crosslinks are responsible for the *E*_a_ increase. After photocrosslinking reactions, stronger intermolecular interactions between collagen molecules appear and they are responsible for the activation energy increase with an increasing shear rate. Thus, the collagen solution after 15 min of UV irradiation was more sensitive to temperature changes and the apparent viscosity decreased more. However, it should be emphasized that structural changes in collagen caused by UV irradiation depend on collagen type, its degree of hydration, and pH conditions [30,31,32]. The results obtained in this study for collagen from *Silver Carp* skin may differ from results obtained for other types of collagen extracted from different sources.

## 4. Conclusions

The results showed that the solution of collagen extracted from the *Silver Carp* fish skin used in this study is a rheologically stable fluid in which the rheological properties do not change over time. The rise in temperature from 30 to 32 °C caused a large reduction of the apparent viscosity value showing that collagen molecules are fully denatured at 32 °C. UV-treatment of collagen solution leads to the increase of the viscosity in the beginning, but after prolonged UV-treatment a large reduction of the apparent viscosity value was observed showing that collagen molecules are fully denatured after one hour of UV treatment. After 15 min of UV-irradiation of collagen solution the collagen molecules underwent thermal denaturation at 30 °C.

Collagen extracted from the *Silver Carp* fish skin is sensitive to UV-irradiation and may undergo physical crosslinking and/or photodegradation. The short time of UV-irradiation may lead to crosslinking of collagen molecules. Collagen extracted from the *Silver Carp* skin can be used in cosmetics and food, however, the products should be stored in a temperature lower than denaturation temperature of collagen. Sterilization of collagen gels with UV light can be done only for a very short time, as prolonged UV treatment leads to photochemical destruction of the collagen molecule.

## Figures and Tables

**Table 1:** The rheological parameters from the Ostwald de Waele model and Cross model for collagen solution as function of concentration and temperature.

T (°C)	Ostwald de Waele Model			Cross Model				
	*n*	*k* (Pas^n^)	R^2^	*η_0_* (Pas)	*η_∞_* (Pas)	*λ* (s)	*m*	R^2^
collagen c = 5 mg/mL								
15	0.39	3.18	0.999	0.673	0.0443	0.0185	1.13	0.999
18	0.39	3.00	0.999	0.657	0.0420	0.0225	1.09	0.999
20	0.39	2.88	0.999	0.582	0.0446	0.0148	1.15	0.999
25	0.41	2.52	0.999	0.538	0.0410	0.0175	1.12	0.998
30	0.43	1.76	0.999	0.362	0.0382	0.00980	1.21	0.998
31	0.48	0.87	0.999	0.184	0.0331	0.00292	1.43	0.997
collagen c = 10 mg/mL								
16	0.25	19.1	0.998	3.079	0.126	0.0225	1.18	0.998
18	0.26	17.5	0.998	3.272	0.286	0.761	0.38	1.00
20	0.27	16.5	0.999	2.742	0.263	0.0101	1.41	0.998
25	0.26	16.3	0.999	2.867	0.101	0.0286	1.13	0.998
30	0.22	7.45	0.963	0.629	0.070	7.69 × 10^−4^	1.80	0.994

**Table 2:** *E*_a_ value for collagen solution at different shear rates.

c (mg/mL)	*E*_a_ (kJ/mol)							
	0 s^−1^	R^2^	43.3 s^−1^	R^2^	90.9 s^−1^	R^2^	110 s^−1^	R^2^
10	17.1	0.914	13.9	0.975	11.4	0.975	10.9	0.979
	19.5	0.657	10.3	0.943	9.3	0.944	8.7	0.940

**Table 3:** The rheological parameters from Ostwald de Waele model and Cross model for collagen solution after 15 min of UV irradiation versus temperature.

T (°C)	Ostwald de Waele Model			Cross Model				
	*n*	*k* (Pas^n^)	R^2^	*η_0_* (Pas)	*η_∞_* (Pas)	*λ* (s)	*m*	R^2^
collagen c = 10 mg/mL								
20	0.27	58.5	0.998	22.04	0.0605	0.2205	0.84	1.00
25	0.23	61.0	0.997	17.17	0.2083	0.1031	0.99	0.999
30	0.13	4.79	0.835	0.358	0.0313	1.38 × 10^−3^	1.78	0.991

**Table 4:** *E*_a_ value for collagen solution at different shear rates after 15 min of UV irradiation.

c (mg/mL)	*E*_a_ (kJ/mol)							
	0 s^−1^	R^2^	43.3 s^−1^	R^2^	90.9 s^−1^	R^2^	110 s^−1^	R^2^
10	49.8	0764.	41.5	0.801	49.1	0.836	54.2	0.859

## References

[1] Miyata T., Sohde T., Rubin A.L., Stenzel K.H. (1971). Effects of ultraviolet irradiation on native and telopeptide-poor collagen. Biochim. Biophys. Acta.

[2] Hyashi T., Curran-Patel S., Prockop D.J. (1979). Thermal stability of the triple helix of type I procollagen and collagen. Precautions for minimizing ultraviolet damage to proteins during circular dichroism studies. Biochemistry.

[3] Fujimori E. (1985). Changes induced by ozone and ultraviolet light in type I collagen. Bovine Achilles tendon collagen versus rat tail tendon collagen. Eur. J. Biochem..

[4] Kaminska A., Sionkowska A. (1996). The effect of UV radiation on the thermal parameters of collagen degradation. Polym. Degrad. Stab..

[5] Sionkowska A., Kaminska A. (1999). Thermal helix-coil transition in UV irradiated collagen from rat tail tendon. Int. J. Biol. Macromol..

[6] Miles C.A., Sionkowska A., Hulin S.L., Sims T.J., Avery N.C., Bailey A.J. (2000). Identification of an intermediate state in the helix-coil degradation of collagen by ultraviolet light. J. Biol. Chem..

[7] Jariashvili K., Madhan B., Brodsky B., Kuchava A., Namicheishvili L., Metreveli N. (2012). UV-damage of collagen: Insides from model collagen peptides. Biopolymers.

[8] Sionkowska A., Wess T.J. (2004). Mechanical properties of UV irradiated rat tail tendon (RTT) collagen. Int. J. Biol. Macromol..

[9] Vizarova K., Bakos D., Rehakova M., Macho V. (1994). Modification of layered atelocollagen by ultraviolet irradiation and chemical cross-linking: Structure stability and mechanical properties. Biomaterials.

[10] Sionkowska A. (2020). Modification of collagen films by ultraviolet irradiation. Polym. Degrad. Stab..

[11] Sionkowska A. (2006). Flash photolysis and pulse radiolysis studies on collagen Type I in acetic acid solution. J. Photochem. Photobiol. B Biol..

[12] Sionkowska A. (2011). Current research on the blends of natural and synthetic polymers: Review. Prog. Polym. Sci..

[13] Bailey A.J., Paul R.G. (1998). Collagen—Is not so simple protein. J. Soc. Leather Technol. Chem..

[14] Orgel J.P., Miller A., Irving T.C., Fischetti R.F., Hammersley A.P., Wess T.J. (2001). The in situ supermolecular structure of type I collagen. Structure.

[15] Bella J. (2010). A new method for describing the helical conformation of collagen: Dependence of the triple helical twist on amino acid sequence. J. Struct. Biol..

[16] Sionkowska A., Skrzyński S., Śmiechowski K., Kołodziejczak A. (2017). The review of versatile application of collagen. Poly. Adv. Technol..

[17] Jaipan P., Nguyen A., Narayan R.J. (2017). Gelatin-based hydrogels for biomedical applications. MRS Commun..

[18] Zhang J., Duan R., Tian Y., Konno K. (2009). Characterisation of acid-soluble collagen from skin of silver carp (Hypophthalmichthys molitrix). Food Chem..

[19] Knapp V.D.M., Barocas V.H., Moon A.G., Tranquillo R.T. (1997). Rheology of reconstituted type I collagen gel in confined compression. J. Rheol..

[20] Lai G., Li Y., Li G. (2008). Effect of concentration and temperature on the rheological behavior of collagen solution. Int. J. Biol. Macromol..

[21] Yang Q., Guo C., Deng F., Ding C., Yang C., Wu H., Ni Y., Huang L., Chen L., Zhang M. (2019). Fabrication of highly concentrated collagens using cooled urea/HAc as novel binary solvent. J. Mol. Liq..

[22] Yang H., Duan L., Li Q., Tian Z., Li G. (2018). Experimental and modeling investigation on the rheological behavior of collagen solution as a function of acetic acid concentration. J. Mech. Behav. Biomed. Mater..

[23] Ghannam M.T., Esmail M.N. (1997). Rheological properties of carboxymethyl cellulose. J. Appl. Polym. Sci..

[24] Lewandowska K. (2007). Comparative studies of rheological properties of polyacrylamide and partially hydrolyzed polyacrylamide solutions. J. Appl. Polym. Sci..

[25] Wang S., Le H., Guo J., Zhao J., Tang H. (2015). Intrinsic viscosity and rheological properties of natural and substituted guar gums in seawater. Int. J. Biol. Macromol..

[26] Tian Z., Duan L., Wu L., Shen L., Li G. (2016). Rheological properties of glutaraldehyde-crosslinked collagen solutions analyzed quantitatively using mechanical models. Mater. Sci. Eng. C.

[27] Martuscelli E. (1984). Melt rheology of a compatible blend: Poly (ethylene oxide)/poly (methyl methacrylate). Die Makromol. Chem. Rapid Commun..

[28] Razavi S.M., Cui S.W., Ding H. (2016). Structural and physicochemical characteristics of a novel water-soluble gum from *Lallemantia royleana* seed. Int. J. Biol. Macromol..

[29] Bertolo M.R.V., Martins V.C.A., Horn M.M., Brenelli L.B., Plepis A.M.G. (2020). Rheological and antioxidant properties of chitosan/gelatin-based materials functionalized by pomegranate peel extract. Carbohydr. Polym..

[30] Xing J.Y., Bai B., Xue W.B., Yang M.Y. (2013). Effect of UV on stability of collagen with consideration of hydratation and fibrillogenesis. Food Sci. Biotechnol..

[31] Mori H., Hara M. (2020). UV irradiation of type I collagen gels changed the morphology of the interconnected brain capillary endothelial cells on them. Mater. Sci. Eng. C.

[32] Zheng X.J., Pei Y., Liu J., Wang K., Tang K.Y. (2017). Effect of UV irradiation on the properties of goatskin collagen matrices. J. Soc. Leather Technol. Chem..

